# Clinical impact of duodenal pancreatic heterotopia – Is there a need for surgical treatment?

**DOI:** 10.1186/s12893-017-0250-x

**Published:** 2017-05-08

**Authors:** Alexander Betzler, Soeren T. Mees, Josefine Pump, Sebastian Schölch, Carolin Zimmermann, Daniela E. Aust, Jürgen Weitz, Thilo Welsch, Marius Distler

**Affiliations:** 1 Department of General, Thoracic and Vascular Surgery, University Hospital Carl Gustav Carus, TU Dresden, Fetscher Str. 74, 01037 Dresden, Germany; 2 Institute for Pathology, University Hospital Carl Gustav Carus, TU Dresden, Fetscher Str. 74, 01307 Dresden, Germany

**Keywords:** Pancreatic heterotopia, Pancreatic resection, Heinrich’s classification, Pancreatic cancer, Chronic pancreatitis

## Abstract

**Background:**

Pancreatic heterotopia (PH) is defined as ectopic pancreatic tissue outside the normal pancreas and its vasculature and duct system. Most frequently, PH is detected incidentally by histopathological examination. The aim of the present study was to analyze a large single-center series of duodenal PH with respect to the clinical presentation.

**Methods:**

A prospective pancreatic database was retrospectively analyzed for cases of PH of the duodenum. All pancreatic and duodenal resections performed between January 2000 and October 2015 were included and screened for histopathologically proven duodenal PH. PH was classified according to Heinrich’s classification (Type I acini, ducts, and islet cells; Type II acini and ducts; Type III only ducts).

**Results:**

A total of 1274 pancreatic and duodenal resections were performed within the study period, and 67 cases of PH (5.3%) were identified. The respective patients were predominantly male (72%) and either underwent pancreatoduodenectomy (*n* = 60); a limited pancreas resection with partial duodenal resection (*n* = 4); distal pancreatectomy with partial duodenal resection (*n* = 1); total pancreatectomy (*n* = 1); or enucleation (*n* = 1). Whereas 65 patients (83.5%) were asymptomatic, 11 patients (18.4%) presented with symptoms related to PH (most frequently with abdominal pain [72%] and duodenal obstruction [55%]). Of those, seven patients (63.6%) had chronic pancreatitis in the heterotopic pancreas. The risk of malignant transformation into adenocarcinoma was 2.9%*.*

**Conclusions:**

PH is found in approximately 5% of pancreatic or duodenal resections and is generally asymptomatic. Chronic pancreatitis is not uncommon in heterotopic pancreatic tissue, and even there is a risk of malignant transformation. PH should be considered for the differential diagnosis of duodenal lesions and surgery should be considered, especially in symptomatic cases.

## Background

Pancreatic heterotopia (PH) was first reported by Jean-Schultz in 1729 and is defined as pancreatic tissue without anatomical or vascular connection to the pancreas [1, 2].

The ectopic pancreatic tissue possesses its own duct system and vascular supply [3, 4]. It is mostly found in the upper gastrointestinal tract (GIT), but may occur anywhere in the GIT [5, 6]. Frequent locations are the duodenum (93.6%), stomach (24–38%), jejunum (0.5–27%), and Meckel’s diverticulum (2–6.5%) [7]. The most widespread explanation of the origin of PH is that the ectopic tissue separates itself from the pancreas during embryonic rotation and fusion of the dorsal and ventral pancreatic buds (misplacement theory) [3, 8]. For a clinical understanding of PH it is important to know that all diseases arising in the genuine pancreas can also develop in heterotopic tissue [3, 6, 9, 10].

Among all abdominal surgeries the incidence of PH ranges from 0.25–1.2%, and specific symptoms have not been described until now [11]. Most patients with PH are asymptomatic, and PH is detected incidentally by histological examination of the specimen. Although malignant transformation originating from PH is extremely rare, it has been reported in several cases in the literature [12, 13]. Because of the scarcity of symptomatic PH cases in the literature [6, 14–16], we investigated our series of duodenal PH with a special focus on its clinical relevance and impact.

## Methods

**Fig. 1:**
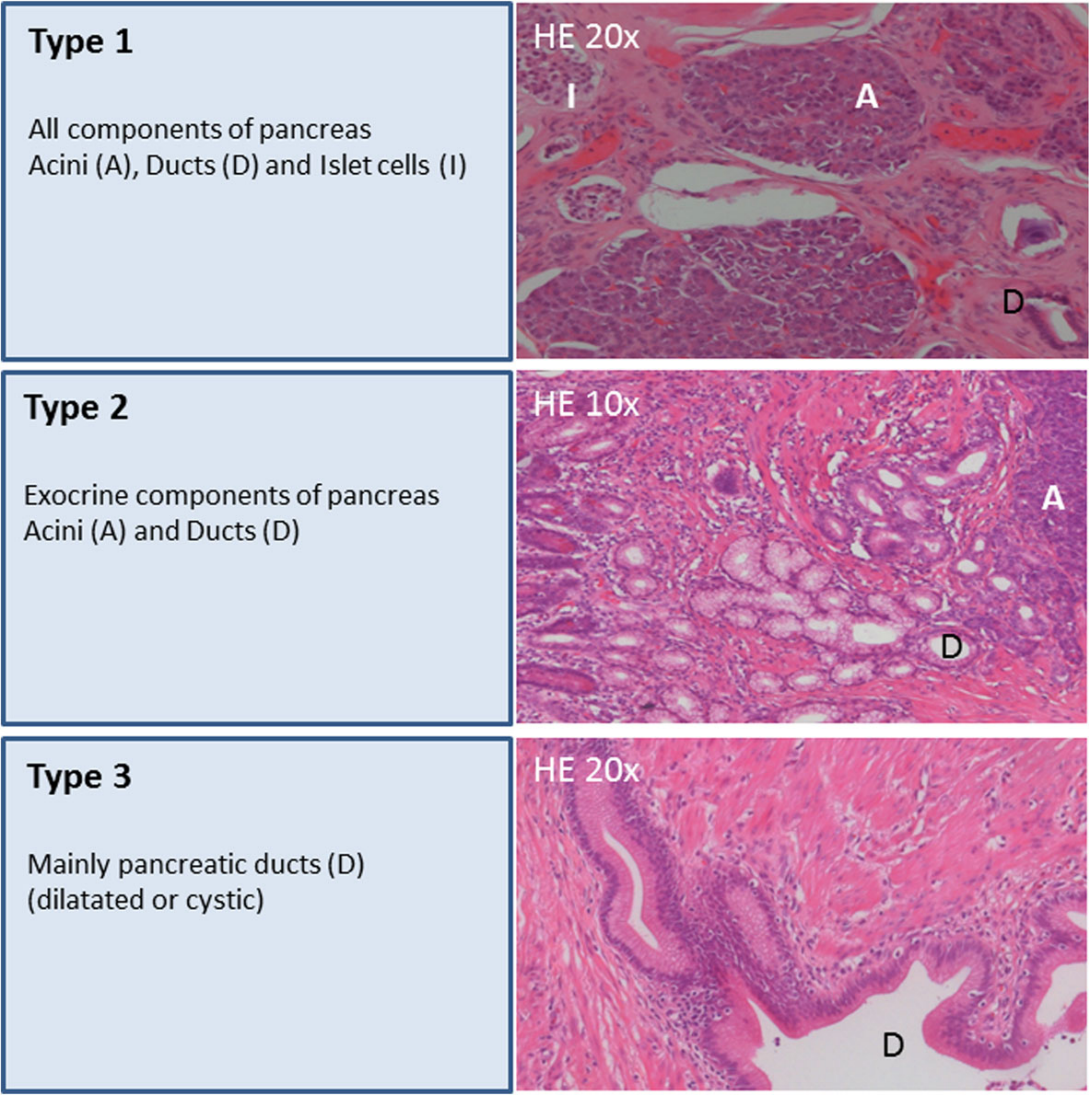
Heinrich’s classification of pancreatic heterotopia

We retrospectively analyzed our prospective pancreatic database for cases with PH of the duodenum. All pancreatic and duodenal resections performed at the Department of Visceral, Thoracic and Vascular Surgery, University Hospital, TU Dresden between January 2000 and October 2015 were included. Partial results have been published elsewhere [17]. Clinical symptoms, surgical procedures and pathological findings were documented for each case. At histological examination, the specimens were stained with hematoxylin and eosin, and a senior GI pathologist (DEA) reviewed each sample regarding the components of pancreatic tissue (including acini, ducts, and islets of Langerhans). PH was classified according to Heinrich’s classification (Fig. 1) [18]. Briefly, PH Type I includes acini, ducts and endocrine islet cells, Type II contains acini and ducts, but no islet cells, and Type III contains only pancreatic ducts.

PH was classified as “symptomatic” if the surgery was directly indicated for PH-associated pathologies, whereas incidental PH diagnosed on postoperative histopathological examination was classified as “asymptomatic”. We compared these two groups regarding type of PH, associated disease and treatment.

In accordance with the guidelines for human subject research, approval was obtained from the ethics committee at the Carl Gustav Carus University Hospital (decision number EK 435102015).

## Results

### Patient cohort

In total, 1274 pancreatic and duodenal resections were performed in our department during the study period. Some 67 cases (5.3%) with histologically proven duodenal PH were identified (19 women and 49 men). The mean age of the whole cohort was 54 years (range 24–76 years). The postoperative histology showed chronic pancreatitis (CP) (*n* = 25; 37.3%), pancreatic ductal adenocarcinomas (PDAC) (*n* = 11; 16.4%), and cystic neoplasms (*n* = 11; 16.4%). Nine operated patients (13.4%) had papillary carcinomas (AP) and six (9%) presented with neuroendocrine tumors (NET). More rare indications for operation included duodenal polyps (two cases; 2.9%), one cholangiocarcinoma, one pancreas divisum, and one duodenal carcinoma (Tables 1 and 2). Pancreatic head resections including pylorus-preserving pancreatoduodenectomies (PPPD) and Whipple procedures represented the vast majority of the operations (*n* = 60; 89.5%). In four cases (5.9%) a segmental pancreatic resection with partial duodenal resection was performed. Furthermore, one patient each underwent a distal pancreatectomy with partial duodenal resection, a total pancreatectomy and an enucleation of the pancreas and the duodenum (1.4%). According to Heinrich’s classification, Type I PH was found in 32 patients (47.9%), Type II in 28 patients (41.7%), and Type III in 7 patients (10.4%) (Tables 1 and 2).

**Table 1:** Characteristics of “asymptomatic patients” with duodenal PH (*n* = 56) (Indication for operation due to clinical presentation and symptoms)

Type of duodenal PH (according to Heinrich)	Age (mean) Sex (m/f)	Operation	Clinical presentation and symptoms^a^	Histology
Type 1 *n* = 26	57.4 years (16/10)	1xpancreatectomie; 3xWhipple; 19× PPPD; 2× partial duodenal resection; 1× enucleation of the pancreas and the duodenum	11xobstructive jaundice 9xepigastric pain 6xrecurrent pancreatitis duodenal stenosis 5xloss of weight recurrent hypoglycemia acid reflux	SCN 5xPDAC 5xIPMN 6xCP 5xAP 4xNET
Type 2 *n* = 24	55.9 years (18/6)	19xPPPD 4xWhipple Pancreas left resection	9xrecurrent pancreatitis 7xobstructive jaundice 10xepigastric pain diarrhoe 3xdilatation pancreas duct 2xpseudocyst 2xduodenal stenosis 5xweight loss gastric stenosis 2xcholestasis	4xIPMN Carcinoma of the bile duct 3xAP 9xCP 2xNET 4xPDAC SCN
Type 3 *n* = 6	57.2 years (4/2)	5xPPPD 1xWhipple	2xrecurrent pancreatitis; 3xobstructive jaundice; 3xepigastric pain; 1xpseudocyst; 1xcystic tumor; 1xampullary tumor; 1xduodenal stenosis; 1× dilatation pancreatic duct	1xPDAC; 3xCP; 1xAP; 1xpancreas divisum

^a^multiple answers possible;

*PDAC* pancreatic ductal adenocarcinoma, *CP*chronic pancreatitis, *AP*ampullary carcinoma, *SCN* serous cystic neoplasia, *IPMN* intraductal papillary mucinous neoplasia, *NET* Neuroendocrine tumor

**Table 2:** Characteristics of “symptomatic patients” with lesions originating from duodenal PH or symptoms due to duodenal PH (*n* = 11)

Type of duodenal PH (according to Heinrich)	Age (mean) Sex (m/f)	Operation	Clinical presentation and symptoms^a^	Histology
Type 1 *n* = 6	58.6 years (5/1)	3xWhipple; 3xPPPD	3xduodenal stenosis; 4xepigastric pain; 1xnausea/vomiting; 1xobstructive jaundice; 1xweigth loss; 1xpseudocysts	3xCP in PH; 2xPDAC in PH; 1× PH in duodenal wall
Type 2 *n* = 4	47.8 years (4/0)	2xpartial duodenal resection; 1xWhipple; 1xPPPD	3xduodenal stenosis; 3xepigastric pain; 1xNausea/vomiting; 1xobstructive jaundice; 1xpseudocysts	1× PH in duodenal wall; 3xCP in PH
Type 3 *n* = 1	47.0 years male	PPPD	recurrent epigastric pain	CP in PH

^a^multiple answers possible

*PDAC* pancreatic ductal adenocarcinoma, *CP* chronic pancreatitis, *PH* pancreatic heterotopia

### “Asymptomatic” subgroup

Fifty-six of the 67 patients (83.5%) were classified as asymptomatic. Performed operations, and postoperative histology are shown on Table 1. In this subgroup the following types of heterotopia were diagnosed: Type I, *n* = 26; Type II, *n* = 24; and Type III, *n* = 6. The patients in the “asymptomatic” cohort presented with the following complaints of the underlying non-PH associated disease (e.g., pancreatic malignancy or chronic pancreatitis): obstructive jaundice, upper abdominal pain, vomiting/nausea, weight loss or duodenal obstruction (Table 1).

### “Symptomatic” subgroup

In 11 cases (16.4%) duodenal PH was responsible for the clinical symptoms (symptomatic subgroup) (Table 2). In this subgroup Heinrich’s Type I and II were predominantly found (Type I *n* = 6; Type II *n* = 4 and Type III *n* = 1). Interestingly, the most frequent PH-related symptom was upper abdominal pain (*n* = 8, 72%), and duodenal obstruction (*n* = 6, 55%). The postoperative histological examination of the symptomatic patients revealed chronic pancreatitis in 7 patients (64%), PDAC in two patients (18%), and duodenal tumors in two cases (18%) originating from the existing duodenal PH. There was no difference in age, sex and type of surgery between the two groups, and there was no significant difference regarding related disease or Heinrich type (*p* > 0.05) between the symptomatic and asymptomatic cases (Table 3).

**Table 3:** Comparison of the asymptomatic and symptomatic subgroups

	Asymptomatic Subgroup *n* = 56	Symptomatic Subgroup *n* = 11	*p*-value
Mean age y (±SD)	56,7 (12.5)	53,6 (12.3)	*p* = 0.45
Sex (m/f)	38/18	10/1	*p* = 0.12 (X^2^ = 2.405)
Type of surgery (n=)			
PPPD	*n* = 43	*n* = 5	*p* = 0.10 (X^2^ = 4.515)
Whipple	*n* = 8	*n* = 4	
Other	*n* = 5	*n* = 2	
Associated disease (*n*=)			
PDAC	*n* = 10	*n* = 2	*p* = 0.05 (X^2^ = 10.47)
CP	*n* = 18	*n* = 7	
Cystic neoplasia	*n* = 11	*n* = 0	
NET	*n* = 6	*n* = 0	
AP	*n* = 9	*n* = 0	
Other	*n* = 2	*n* = 2	
Heinrich Type			
I	26	6	*p* = 0.88 (X^2^ = 0.2428)
II	24	4	
III	6	1	

*PDAC* pancreatic ductal adenocarcinoma, *CP* chronic pancreatitis, *NET* neuroendocrine tumor

## Discussion

PH is described as a rare pathological entity, and a preoperative clinical diagnosis is difficult because characteristic clinical symptoms are frequently camouflaged by the multitude of underlying diseases [3, 6, 19]. Clinical series are rare and most data in the literature stem from case reports.

The present study focused on duodenal PH. Approximately 5% of the patients undergoing pancreatic and/or duodenal resections in our cohort were diagnosed with PH, and thus PH was not a particularly rare finding. Our study confirms that most of the patients with PH were asymptomatic, and therefore PH was discovered incidentally. However, depending on its location and diameter, heterotopic pancreatic tissue can lead to nonspecific symptoms [20–22]. According to the literature, lesions are more likely to be symptomatic if they are >2 cm in diameter [23]. This seems to be especially true for tumors that are located in the duodenum due to the anatomic character of this region of the digestive tract. Nevertheless, due to a lack of data concerning the diameter of the duodenal PH-lesions we could not make a clear statement to this point. But abdominal pain is the most common –but nonspecific– symptom of pancreatic heterotopia, [4] as found in the present study (73%). Consequently, the nonspecific set of symptoms makes the clinical diagnosis of PH challenging; none of the patients in the present analysis was diagnosed with PH preoperatively.

### Differential diagnosis of duodenal heterotopia

In general, PH lesions in the GIT are detectable by endoscopy. PH often presents as a submucosal swelling covered by normal mucosa and can easily be mistaken as gastrointestinal stroma tumor (GIST) or leiomyoma using endoscopy, ultrasonography or CT scanning [24]. In addition the risk of false negative biopsy results is high because ectopic tissue is most commonly located in the submucosal layer (76%), and sporadically appears in the muscular layer (15%), or in the subserosa (9%) [25]. Therefore, most biopsies are inconclusive, because of inadequate tissue samples [22]. In this context, endoscopic ultrasound-guided fine-needle aspiration (EUS-FNA) has been found to be valuable in the diagnosis of upper GIT lesions [26, 27].

**Fig. 2:**
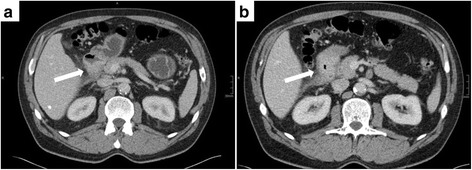
Computed tomography (CT) scans (**a**/**b**) of a duodenal pancreatic heterotopia (arrows) with a duodenal stenosis

In the present study, endoscopic ultrasound was not one of the standard preoperative investigations but the lesions were differentiated by preoperative CT and/or MRI scans (Fig. 2). If one looks on the presented CT-scan of a duodenal PH, it was especially difficult to distinguish the tumor from the original pancreas because of the close proximity of the two organs (Fig. 2). Based on current data on the value of EUS in the diagnosis of upper GIT lesions, EUS should be performed if a submucosal lesion is suspected. From the clinical point of view it is often impossible to distinguish GIST, lymphomas, peptic ulcer disease, or malignancies from heterotopic pancreatic tissue [16, 20, 22]. To diagnose PH, histopathological examination is therefore essential.

### Malignant transformation of pancreatic heterotopia

Several studies have demonstrated that any disease of the ordinary pancreas can also arise in the heterotopic tissue, such as acute and chronic pancreatitis, the occurrence of pseudocystic changes, or even a malignant transformation to adenocarcinoma or acinar cell carcinoma [3, 6, 10, 16, 28–30]. The present results are in line with these findings, as two out of 67 patients with PH developed adenocarcinoma by malignant transformation of the heterotopic pancreatic tissue (2.9%). Guillou et al. stated that the incidence of malignancy due to heterotopic pancreatic tissue is 0.7% and therefore is extremely rare [13]. They studied the frequency of malignant transformations among 146 cases of PH between 1975 and 1991, including surgical and autopsy specimens. In a study by Makhlouf et al. two out of 109 patients (1.8%) with PH of the gastrointestinal tract were diagnosed with adenocarcinoma between 1970 and 1997 [12]. Malignancy is therefore a differential diagnosis and should be excluded. Furthermore, histopathological examination of the resected specimen of the 11 symptomatic patients in the present study showed chronic pancreatitis in seven cases (63.6%) and a duodenal tumor (adenoma) with no signs of chronic pancreatitis or malignancy in two cases (18%). Interestingly cystic lesions or NET arising from a duodenal PH were not found in our symptomatic subgroup. Furthermore, no specific Heinrich’s type was associated with symptoms or malignancy.

### Management of PH

For patients with symptomatic PH, local resection of the lesion seems to be the most appropriate therapy [16]. Patients underwent partial duodenal resections in two cases due to a symptomatic PH with suspicion of a duodenal tumor after intraoperative exclusion of malignancy by frozen section. Although endoscopic therapy is currently being evaluated for removal of ectopic pancreatic tissue, surgery remains the standard therapy [31, 32]. If histologically proven PH is asymptomatic and malignancy is definitely excluded, it can be treated conservatively. Nevertheless, if PH is found incidentally during a surgical procedure, excision should be considered due to its potential for becoming symptomatic and malignant. If malignancy is suspected extended oncological surgical resection (e.g., PPPD) is justified. The prognosis of patients with adenocarcinoma arising from PH seems to be better compared to patients with tumors arising from the pancreas itself, probably due to earlier presentation [16, 33].

## Conclusion

In summary, PH of the duodenum represents a rare diagnosis and most patients are asymptomatic. Duodenal PH is mostly diagnosed by histological evaluation of surgical specimens resected for different pathologies. Nevertheless, the present results indicate that nearly all diseases of the genuine pancreas can occur in heterotopic pancreatic tissue. Therefore, depending on the current disease, different symptoms can appear and lead to another diagnosis. Ectopic duodenal pancreatic tissue should be considered in the differential diagnosis when a duodenal lesion is detected. Surgical resection is indicated if the lesion is symptomatic or malignancy cannot be excluded.

## Abbreviations

PDAC: Pancreatic ductal adenocarcinoma
NET: Neuroendocrine tumors
GIT: Gastrointestinal tract
PH: Pancreatic heterotopia
PPPD: Pylorus-preserving pancreatoduodenectomy
EUS-FNA: Endoscopic ultrasound-guided fine-needle aspiration
